# From research to clinical practice: a European neuroradiological survey on quantitative advanced MRI implementation

**DOI:** 10.1007/s00330-020-07582-2

**Published:** 2021-01-22

**Authors:** Elia Manfrini, Marion Smits, Steffi Thust, Sergej Geiger, Zeynep Bendella, Jan Petr, Laszlo Solymosi, Vera C. Keil

**Affiliations:** 1 Department of Neuroradiology, Bonn University Hospital, Venusberg-Campus 1, 53127 Bonn, Germany; 2 Facoltà di Medicina e Chirurgia, Università Politecnica delle Marche, Via Tronto 10, 60126 Ancona, Italy; 3 Department of Radiology and Nuclear Medicine (Ne-515), Erasmus MC, PO Box 2040, 3000 CA Rotterdam, The Netherlands; 4 National Hospital for Neurology and Neurosurgery, University College London Hospitals, London, UK; 5 Department of Brain Rehabilitation and Repair, UCL Institute of Neurology, Queen Square, London, WC1N 3BG UK; 6 Helmholtz-Zentrum Dresden-Rossendorf, Institute of Radiopharmaceutical Cancer Research, Dresden, Germany; 7 Department of Radiology, Section Neuroradiology, Amsterdam University Medical Center, VUmc, Amsterdam, The Netherlands

**Keywords:** Neuroimaging, Perfusion imaging, Magnetic resonance imaging, Surveys and questionnaires

## Abstract

**Objective:**

Quantitative MRI (qMRI) methods provide versatile neuroradiological applications and are a hot topic in research. The degree of their clinical implementation is however barely known. This survey was created to illuminate which and how qMRI techniques are currently applied across Europe.

**Methods:**

In total, 4753 neuroradiologists from 27 countries received an online questionnaire. Demographic and professional data, experience with qMRI techniques in the brain and head and neck, usage, reasons for/against application, and knowledge of the QIBA and EIBALL initiatives were assessed.

**Results:**

Two hundred seventy-two responders in 23 countries used the following techniques clinically (mean values in %): DWI (82.0%, *n* = 223), DSC (67.3%, *n* = 183), MRS (64.3%, *n* = 175), DCE (43.4%, *n* = 118), BOLD-fMRI (42.6%, *n* = 116), ASL (37.5%, *n* = 102), fat quantification (25.0%, *n* = 68), T2 mapping (16.9%, *n* = 46), T1 mapping (15.1%, *n* = 41), PET-MRI (11.8%, *n* = 32), IVIM (5.5%, *n* = 15), APT-CEST (4.8%, *n* = 13), and DKI (3.3%, *n* = 9). The most frequent usage indications for any qMRI technique were tissue differentiation (82.4%, *n* = 224) and oncological monitoring (72.8%, *n* = 198). Usage differed between countries, e.g. ASL: Germany (*n* = 13/63; 20.6%) vs. France (*n* = 31/40; 77.5%). Neuroradiologists endorsed the use of qMRI because of an improved diagnostic accuracy (89.3%, *n* = 243), but 50.0% (*n* = 136) are in need of better technology, 34.9% (*n* = 95) wish for more communication, and 31.3% need help with result interpretation/generation (*n* = 85). QIBA and EIBALL were not well known (12.5%, *n* = 34, and 11.0%, *n* = 30).

**Conclusions:**

The clinical implementation of qMRI methods is highly variable. Beyond the aspect of readiness for clinical use, better availability of support and a wider dissemination of guidelines could catalyse a broader implementation.

**Key Points:**

*• Neuroradiologists endorse the use of qMRI techniques as they subjectively improve diagnostic accuracy.*

*• Clinical implementation is highly variable between countries, techniques, and indications.*

*• The use of advanced imaging could be promoted through an increase in technical support and training of both doctors and technicians.*

**Supplementary Information:**

The online version contains supplementary material available at 10.1007/s00330-020-07582-2.

## Introduction

Quantitative MRI (qMRI) techniques, both technically and with respect to clinical indication, cover a very broad field of applications [1]. While standard MRI techniques classically provide visual-anatomical information [2], quantitative techniques allow an insight into the physiological activity or biochemical composition of the tissue through quantifiable parameters [3, 4]. qMRI techniques comprise a broad range of sequence applications and, mostly in a research setting, have shown benefits on innumerable levels including vascular and neoplastic diseases, neurodegeneration, or infectious and inflammatory brain lesions [5–12].

Based on the long-standing research efforts and increasing availability of user-friendly post-processing software, one should expect a broad application of advanced MRI techniques in clinical practice. While several of the techniques were first proposed several decades ago: DSC [13], DCE [14], IVIM [15], ASL [16], and relaxometry [17], a routine application is recommended only for a limited range of diseases and techniques, such as DWI and DSC in glioma imaging [18]. For many other techniques, such as IVIM or ASL, a clinical routine introduction is still not within close reach. One of the reasons is that methodological standardisation remains low and standards for acquisition and processing are limited [19–21].

In the long term, the routine clinical implementation of innovative qMRI techniques is pivotal to justify future research in the field and its funding. It is, however, very difficult to estimate how far the process of clinical implementation has advanced without conducting a wider investigation.

The aim of this European survey was to find out which, how, and to what extent qMRI techniques are applied to solve neuroradiological questions in a primarily clinical setting. The in-depth analysis also focuses on the reasons for the lack of clinical application and general knowledge of qMRI.

## Materials and methods

### Questionnaire

The online questionnaire had a total of 13 main questions to be answered as free text, or multiple, dichotomous, and single-choice answers (online supplement 1). Google Forms was used to implement the questionnaire (Google Inc.). To enhance clarity, techniques that provide quantitative morphometric measurements but are based on conventional MRI sequences were not included in this survey. Brain and head/neck were surveyed as separate organ systems.

The survey was anonymous, voluntary without incentives and all responses were treated confidentially. Information on the country of work and categorisation of the employing institution were mandatory. By design, it was therefore impossible to reliably identify multiple answers from a single institution and thus determine the exact number of institutions answering. Furthermore, respondents could decide if their institution is classified as a large or small hospital.

### Questionnaire distribution

The questionnaire was disseminated in English, German, Italian, Spanish, French, Turkish, Russian, and Portuguese. The questionnaire was emailed to 27 European countries and Russia, Turkey, and Israel as listed in detail in the online supplement 2.

**Fig. 1:**
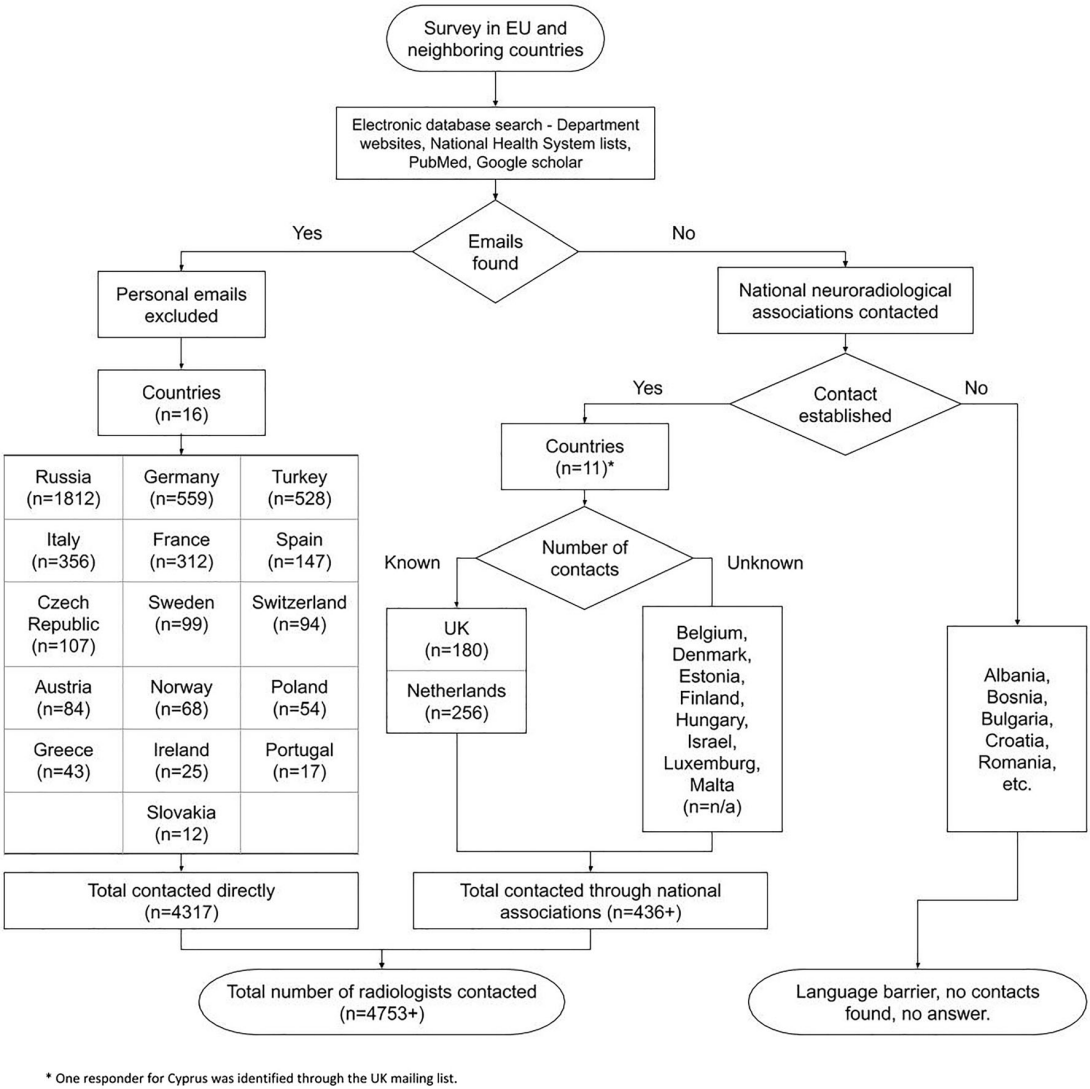
Flow diagram illustrating the data acquisition process

The exact contact procedure is described in the online supplement 3 and technically by Fig. 1 (Fig. 1, online supplement 3). The questionnaire was open from Mid-July 2019 to Mid-October 2019. Invitations were sent out three times. Additional phone interviews were conducted in German-speaking countries dedicated to the locally large number of radiologists working in outpatient practices.

### Survey analysis

Numerical analyses were performed in Microsoft Excel. Fisher’s exact tests were performed with SPSS® V. 26.0 (IBM Corp.) to identify significant differences between groups where applicable.

Answers from professionals who had multiple workplaces were included, but only their primary working place was considered. It was possible that more than one radiologist affiliated with the same institution would fill in and submit the questionnaire, or that the same participant would reply more than once. Therefore, answers were screened for probable redundancies.

If someone denied the use of a certain technique in question 3, but later reported in the detailed answer block (question 6) that she/he used it for several indications, we extrapolated that the responder indeed used the technique, but erroneously forgot to tick the box in the beginning. In the reverse case (with the detailed answers left blank), a non-intentional blank was presumed, e.g. due to oversight.

As advanced MRI needs extra processing and scanning time, we investigated the association between the potential dissemination of knowledge and the presumed economic constraints. qMRI technique dissemination was analysed based on the gross domestic product per person (GDP pP) and the research and development expenditures per country as percentage of GDP (GERD) separating countries of respondents into above or below EU 28 average [22].

## Results

### Demographic information of respondents

**Fig. 2:**
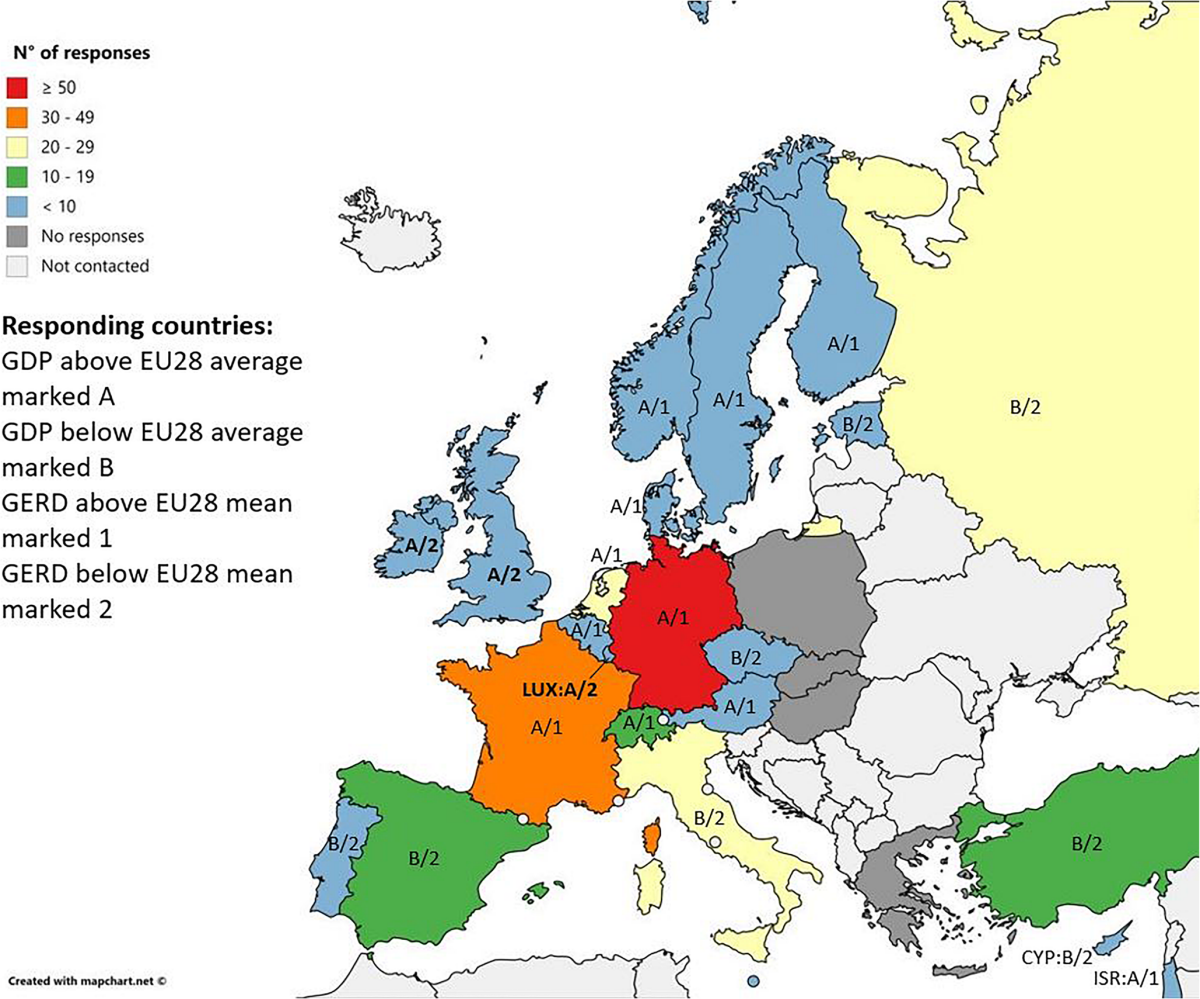
Map showing the number of radiologists responding by country with GDP and GERD. GDP, gross domestic product per capita in 2018; GERD, gross domestic expenditure on research and development as percentage of GDP (in 2018 except for Switzerland with last numbers from 2017); in bold are countries with discrepancy between economic power and spending on research (“under average spenders”)

In total, 272 neuroradiologists answered in 23 countries (online supplement 2, Fig. 2). The average return rate per country was 6.7 ± 6.1% (range from 0.0 to 23.5%) of the respondents. The following countries had zero returns: Greece, Slovakia, Hungary, and Poland, or a return rate of < 2%: Russia (1.2%, *n* = 22/1812) and Norway (1.5%, *n* = 1/68).

Most respondents worked in institutions of 6 to 20 doctors (44.9%, *n* = 122/272). However, 29.0% (*n* = 79/272) had more than 50 colleagues. Response rates varied by institution type (Table 1).

**Table 1:** Questionnaire response rates by institution type

Type	Response rate (in % and standard deviation	Range by country (%)
University hospital	19.4 ± 16.3	0.0–60.7
Large hospital	5.9 ± 5.4	0.0–15.4
Small hospital	2.6 ± 3.2	0.0–10.3
Outpatient practice	0.8 ± 1.9	0.0–6.7
Teleradiology centres	0.4 ± 0.7	0.0–1.8
Research institution	0.7 ± 1.3	0.0–3.4

### Usage dissemination by sequence and indication

The most commonly applied qMRI sequence based on question 3 was DWI (82.0%), followed by DSC (67.3%) and MRS (64.3%). DCE, BOLD-based techniques, ASL, and fat quantification still had an intermediate dissemination of 43.4%, 42.6%, 37.5%, and 25.0%, respectively. T1 and T2 mapping, PET-MR, IVIM, APT-CEST, and diffusion kurtosis imaging (DKI) were uncommon (15.1%, 16.9%, 11.8%, 5.5%, 4.8%, and 3.3%, respectively) in most institutions.

Sequence usage showed extensive geographical differences (online supplements 4 to 9).

For the detailed clinical indication questions (section II, question 6 of the questionnaire), DWI was not an answer option. A total of 94.9% of respondents used at least one qMRI technique other than DWI in the brain, while only 31.3% applied qMRI sequences in head and neck MRI.

Tissue differentiation (82.4%) and oncological monitoring (72.8%) were the most common reasons to apply any quantitative technique. qMRI (other than DWI) was less common for stroke imaging (58.8%) and only a minority of respondents used it for neurodegenerative diseases (26.1%) or multiple sclerosis (22.8%).

The most frequently applied techniques for glioma imaging were DSC (73.2%) and MRS (54.8%). DSC (39.3%) and ASL (20.6%) had an intermediate use in stroke diagnostics and oncological monitoring. PET-MRI and APT-CEST were rarely used (10.7% and 0.0%, for general oncological monitoring; 9.6% and 2.2% for glioma diagnostics; 8.1% and 1.1% in lesion differentiation, respectively).

In the head and neck region, lesion differentiation was the single most common reason to apply quantitative techniques, with and DSC (29.0%) or DCE (20.6%) most frequently used (DWI was not an answer option).

### Dissemination by institution type and GDP

Clear trends could be observed between the type of institution and the frequency of use of a qMRI technique based on the compulsory country answer and answers to questions 3 of the questionnaire. University hospitals were the most frequent users of a technique, large hospitals second, and small hospitals the least likely users. With DSC as an example, university hospitals used it more often than large hospitals (126/156 vs. 46/70; *p* = 0.01), or small hospitals (13/36; p = 0.01).

The countries with GDP above the EU28 average in 2018 (44,748 USD/capita) used the following techniques significantly more than the countries below this average: DSC (*p* = 0.0001), ASL (*p* = 0.02), DWI (*p* = 0.0001), CEST (*p* = 0.04), T2 mapping (*p* = 0.001), and MRS (*p* = 0.003). DSC (*p* = 0.0007), DWI (*p* = 0.0001), T2 mapping (*p* = 0.004), and MRS (*p* = 0.002) were significantly more often performed in countries with an above-average EU28 GERD (2.03% of GDP; Fig. 2).

### Motivation analysis and network knowledge

**Fig. 3:**
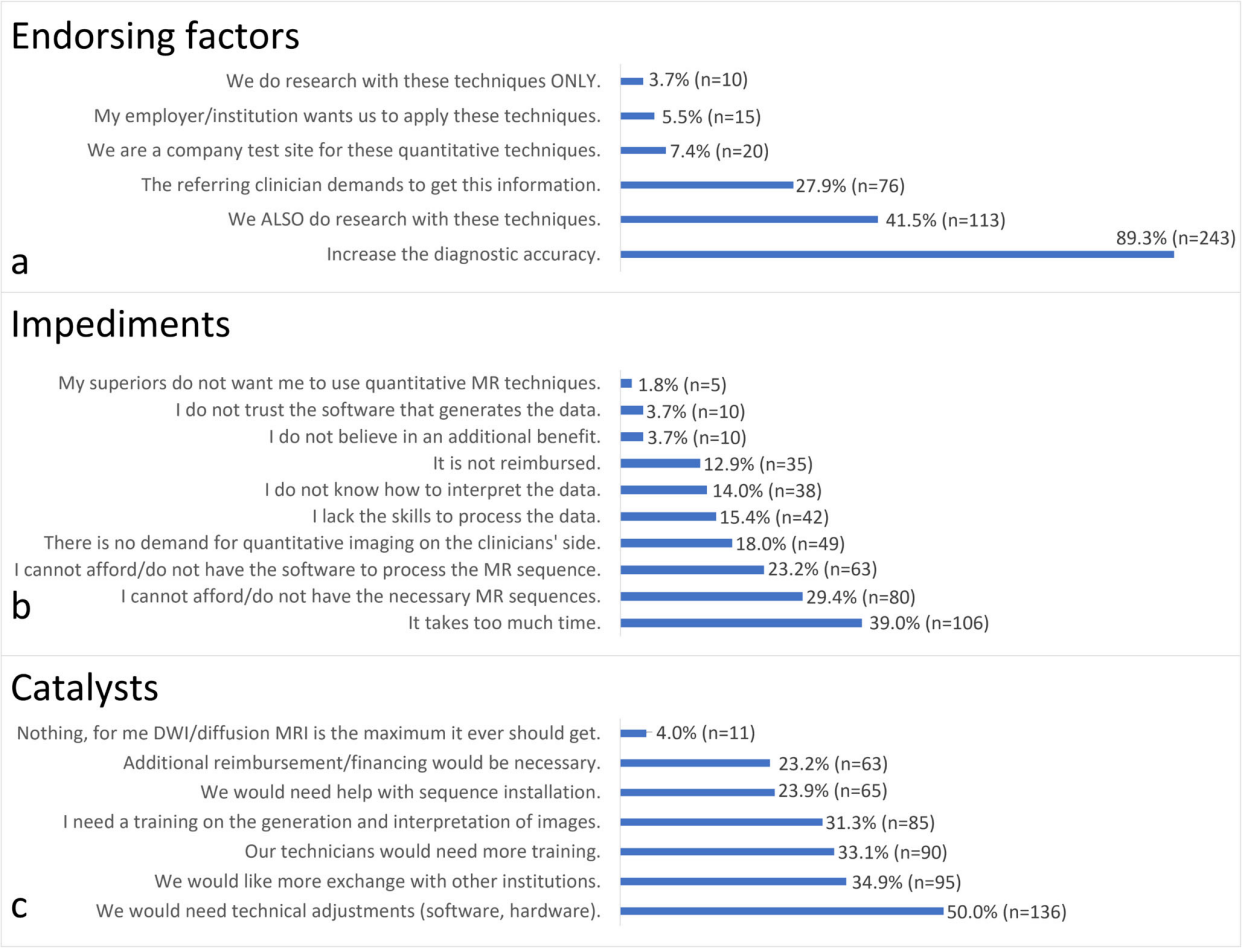
Factors influencing the use of quantitative advanced MRI sequences in clinical practice. **a** Factors that endorse the use of these quantitative MRI sequences. **b** Factors that impede the use of these quantitative MRI sequences. **c** Factors that could catalyse a greater implementation

Figure 3 illustrates the main reasons for and against the use of qMRI based on section III, questions 7 to 9. In summary, the majority of neuroradiologists favoured the usage of qMRI because of an improved diagnostic accuracy (89.3%). Only a small minority (4.0%) did not see any advantages in qMRI.

Scientific reasons (41.5%) were a major impulse to perform additional quantitative sequences.

The greatest impediment for advanced MRI applications seemed a lack of time (39.0%) rather than a lack of financial compensation (12.9%).

Notably, both QIBA and EIBALL as imaging biomarker institutions were not widely known (12.5% and 11.0%, respectively) amongst clinicians.

## Discussion

This survey is unique in its purpose and aimed to assess the clinical dissemination of qMRI techniques in neuroradiological practice across Europe. While common usage of DWI, DSC, and MRS was confirmed for certain indications such as glioma imaging, it is apparent that some techniques are rarely used, show variable use by country, or are only performed for a limited number of indications. Our data show that an overwhelming majority of respondents sees a benefit in the use of qMRI for their diagnostic work, but mention a lack of time as the main reason not to implement qMRI techniques. This factor, together with the need for more training, technical adjustments, and an improved exchange of expertise with other institutions, was identified through this survey as the key element hampering the clinical translation of qMRI into clinical neuroradiology.

In most European countries, MRI protocols are being continuously shortened to reduce waiting times for MRI. These waiting times differ largely between countries from an average of 18 days in the Netherlands [23] to 126 days in Ireland [24] and show regional differences within countries (e.g. Italy, North-East 50 days vs. South 111 days [25]). Beyond protocol length, waiting times depend on several factors: number of available MRI scanners, radiologists, and limitations of healthcare budgets [26]. While, e.g., the UK faces a bottleneck for qMRI implementation regarding all of these factors, reasons for limitations of qMRI are different in Germany and have more than four times as many MRI scanners per inhabitant as the UK (37/1M vs. 9/1M inhabitants) [27]. In Germany, insurance compensation frequently has a fixed price per scan without sufficient compensation for additional sequences, which may limit the incentive to add qMRI. This may partially explain the relatively lower usage of many techniques in Germany compared to other above-average GDP and GERD countries, e.g. France—a country with also relatively many respondents, but fewer scanners (14/1M inhabitants; online supplements 5–9). The larger number of scanners in Germany is also not sufficiently reflected in the number of exams performed: 143 MRI exams/1000 inhabitants/year in Germany as opposed to UK and France with 62 and 114, respectively, which makes the possibly lower use of qMRI techniques even more surprising.

Respondents in most countries already use some kind of qMRI technique at least for some indications according to our results. We therefore interpret their claims of impediments and incentives for qMRI as a wish for more extensive use. The questionnaire responses show directions on how to allow qMRI to find a larger entrance into clinical neuroradiology. The level of evidence concerning a diagnostic benefit must be increased, as this is the key to acceptance of a technique into guidelines and eventually financing by the public sector, which is needed to cover costs for technical adjustments, software, and training. DSC in glioma imaging, which is now part of EORTC guidelines, is an example. It had the highest prevalence as an indication in this study and was previously identified as relevant in other surveys on either glioma or perfusion imaging [18, 28, 29]. For some techniques, such as ASL, which is much less used despite reduced risks for the patient and reduced costs compared to DSC, clinical research should possibly be facilitated. Another aspect is the clinical indications for which qMRI techniques. A large discrepancy can be observed between scientific trials and clinical implementation, e.g. concerning neurodegenerative diseases as also testified in this survey. In many countries, patients are still likely to receive a CT scan when dementia is suspected. Although neurodegenerative diseases and also respective imaging receive a lot of funding, there is currently limited evidence to justify qMRI technique implementation. DWI imaging and structural brain volumetric analysis mark the quantitative imaging aspect in this field, as corroborated by very recent clinical data regarding dementia imaging in Europe [30].

In this context, and suggested by our data, one major obstacle to implement qMRI sequences is not a lack of acceptance by clinicians, but indeed a multi-level shortfall of clinical technical skill. Our analyses by institution type uncovered important associations with the likeliness to use qMRI techniques. A clear slope of dissemination was observed from university setting already to large hospitals, and further to small sites. Only DWI would be available at all types of sites in the majority of cases, with all other qMRI techniques mark the exception rather than the norm outside a university setting. This corroborates the slow velocity of trickle-down effects. Therefore, beyond time constraints and financial burdens, clinicians in smaller institutions are also in the need of better knowledge transfer as a motivation for implementation. Here, not only scientists but also vendors are required to act through hands-on trainings at a low financial and knowledge threshold. The involvement of non-university sites in scientific projects can be another meaningful way to accelerate clinical dissemination of qMRI techniques. An example can be Denmark, which integrates smaller hospitals into large national trials and facilitates also the implementation of private-public partnership projects [31]. Such advances must however be supported by an interaction of the national- and European-level political forces of both the healthcare and science sectors. Here, institutions such as the ESR and in particular EIBALL could act as important lobbyists, but must still be better known according to our data and assuming an over-average interested group of Neuroradiologists as respondents. The radiological training curriculum has the potential to be extended concerning advanced imaging data processing and interpretation. One should remember that many countries do not provide a strictly hierarchical structure of primary to tertiary healthcare providers. Small institutions can, therefore, also be confronted with complex cases that may benefit from qMRI.

A worrying revelation of this study is the possible association between qMRI usage and GDP as well as GERD. Living in a lower GDP European country negatively affects the patients’ chances to receive a neuroradiological examination that includes DSC and DWI—two qMRI methods, which are considered an important part of glioma MRI protocols [32, 33]. While political solutions to achieve the desired equal standards of European healthcare are one aspect, neuroradiological societies and scientific European initiatives can contribute their share, too, e.g. through knowledge exchange and provision of free software solutions.

This study has a few limitations starting with a selection bias due to the variable modes of contact to the radiologists. Only a proportion of radiologists were contactable in every country with university centres being, partially deliberately, overrepresented. The resulting data distortions reduce the representativeness of the survey data. Another aspect is the uneven response rate. One reason could be the mode of communication that may have excluded, some respondents, e.g. due to language barriers. Furthermore, it must be assumed that despite the anonymous nature of the survey, respondents may not have felt comfortable providing realistic answers. They may have also mixed up a clinical implementation with research implementation performed in a clinical setting, e.g. an experimental CEST sequence as part of a clinical programme. Neuroradiologists frequently working with quantitative techniques were probably also more willing to answer the survey, biasing results towards a wider use. There remains minimal survey data on the topic, and this survey is unique in its focus. It served as a first attempt to clarify the extent of the current clinical use of qMRI in neuroradiology in Europe and can, also due to the size, not be considered fully representative. The additive value of qMRI techniques must be explored in prospective blinded comparative studies elsewhere and was not attempted to be answered within this survey.

### Conclusion

Usage of qMRI techniques in neuroradiology is not standardised throughout Europe. Its clinical translation varies substantially between techniques as well as geographically. Local healthcare policies and variable sharing of expertise can be presumed as underlying reasons, while neuroradiologists in principle feel positive about qMRI opportunities. This survey highlights an unmet need to promote qMRI through larger clinical studies showing a convincing benefit, improved networking between clinicians and scientists as well as training.

## Supplementary information

ESM 1 (DOCX 8445 kb)

## Abbreviations

ADC: Apparent diffusion coefficient
APT: Amide-proton transfer
ASL: Arterial spin labelling
BOLD: Blood-oxygen level dependent (imaging)
CEST: Chemical exchange saturation transfer
DCE: Dynamic contrast-enhanced
DKI: Diffusion kurtosis imaging
DSC: Dynamic susceptibility contrast
DTI: Diffusion tensor imaging
DWI: Diffusion-weighted imaging
EIBALL: European Imaging Biomarker Alliance
FatQuant: Fat quantification techniques
fMRI: Functional MRI
GDP: Gross domestic product
GERD: Gross domestic expenditure on research and development as percentage of GDP
IVIM: Intravoxel-incoherent motion
MRI: Magnetic resonance imaging
MRS: Magnetic resonance spectroscopy
QIBA: Quantitative Imaging Biomarkers Alliance
qMRI: Quantitative magnetic resonance imaging

## References

[1] Carnevale L, Lembo G (2019) Innovative MRI techniques in neuroimaging approaches for cerebrovascular diseases and vascular cognitive impairment. Int J Mol Sci 20(11). 10.3390/ijms20112656 10.3390/ijms20112656 PMC6600149 31151154

[2] Lerch JP, van der Kouwe AJ, Raznahan A (2017). Studying neuroanatomy using MRI. Nat Neurosci.

[3] Pope WB, Djoukhadar I, Jackson A (2016). Neuroimaging. Handb Clin Neurol.

[4] Chen JJ (2019). Functional MRI of brain physiology in aging and neurodegenerative diseases. Neuroimage.

[5] Bandettini PA (2012). Twenty years of functional MRI: the science and the stories. Neuroimage.

[6] Meijer FJ, Goraj B (2014). Brain MRI in Parkinson’s disease. Front Biosci (Elite Ed).

[7] Giorgio A, De Stefano N (2016). Advanced structural and functional brain MRI in multiple sclerosis. Semin Neurol.

[8] Widmann G, Henninger B, Kremser C, Jaschke W (2017). MRI sequences in head & neck radiology - state of the art. Rofo.

[9] Shukla G, Alexander GS, Bakas S (2017). Advanced magnetic resonance imaging in glioblastoma: a review. Chin Clin Oncol.

[10] Yousaf T, Dervenoulas G, Politis M (2018). Advances in MRI methodology. Int Rev Neurobiol.

[11] Bonm AV, Ritterbusch R, Throckmorton P, Graber JJ (2020). Clinical imaging for diagnostic challenges in the management of gliomas: a review. J Neuroimaging.

[12] Fujima N, Kameda H, Shimizu Y (2020). Utility of a diffusion-weighted arterial spin labeling (DW-ASL) technique for evaluating the progression of brain white matter lesions. Magn Reson Imaging.

[13] Edelman RR, Mattle HP, Atkinson DJ (1990). Cerebral blood flow: assessment with dynamic contrast-enhanced T2*-weighted MR imaging at 1.5 T. Radiology.

[14] Tofts PS, Kermode AG (1989). Blood brain barrier permeability in multiple sclerosis using labelled DTPA with PET, CT and MRI. J Neurol Neurosurg Psychiatry.

[15] Le Bihan D (1988). Intravoxel incoherent motion imaging using steady-state free precession. Magn Reson Med.

[16] Williams DS, Detre JA, Leigh JS, Koretsky AP (1992). Magnetic resonance imaging of perfusion using spin inversion of arterial water. Proc Natl Acad Sci U S A.

[17] Damadian R (1971). Tumor detection by nuclear magnetic resonance. Science.

[18] Thust SC, Heiland S, Falini A (2018). Glioma imaging in Europe: a survey of 220 centres and recommendations for best clinical practice. Eur Radiol.

[19] Alsop DC, Detre JA, Golay X (2015). Recommended implementation of arterial spin-labeled perfusion MRI for clinical applications: a consensus of the ISMRM perfusion study group and the European consortium for ASL in dementia. Magn Reson Med.

[20] Boxerman JL, Quarles CC, Hu LS et al (2020) Consensus recommendations for a dynamic susceptibility contrast MRI protocol for use in high-grade gliomas. Neuro Oncol. 10.1093/neuonc/noaa141 10.1093/neuonc/noaa141 PMC7523451 32516388

[21] Mutsaerts H, Petr J, Groot P et al (2020) ExploreASL: an image processing pipeline for multi-center ASL perfusion MRI studies. Neuroimage 117031. 10.1016/j.neuroimage.2020.117031 10.1016/j.neuroimage.2020.117031 32526385

[22] OECD (2020) Main science and technology indicators. 2019(2). 10.1787/g2g9ff07-en

[23] Statista (2018) Average waiting time for MRI scans in the Netherlands from 2010 to 2016. Statista. https://www.statista.com/statistics/979147/average-waiting-time-for-mri-scans-in-the-netherlands/. Accessed 06 June 2020

[24] O’Shea MT (2016) Access to diagnostics used to detect cancer. Irish College of General Practitioners. https://www.lenus.ie/handle/10147/615148. Accessed 06 June 2020

[25] Statista (2017) Average waiting time to get an appointment for a magnetic resonance imaging scan in Italy in 2017, by geographic area. Statista. https://www.statista.com/statistics/934110/waiting-time-for-an-mri-by-area-in-italy/. Accessed 06 June 2020

[26] Stat ECE Healthcare resource statistics - technical resources and medical technology. https://ec.europa.eu/eurostat/statistics-explained/index.php/Healthcare_resource_statistics_-_technical_resources_and_medical_technology#Use_of_medical_technology. Accessed 06 June 2020

[27] OECD (2019) Health at a glance: OECD indicators. OECD Publishing. 10.1787/4dd50c09-en. Accessed 06 June 2020 2020

[28] Dickerson E, Srinivasan A (2016). Multicenter survey of current practice patterns in perfusion MRI in neuroradiology: why, when, and how is it performed?. AJR Am J Roentgenol.

[29] Freyschlag CF, Krieg SM, Kerschbaumer J (2018). Imaging practice in low-grade gliomas among European specialized centers and proposal for a minimum core of imaging. J Neurooncol.

[30] Vernooij MW, Pizzini FB, Schmidt R (2019). Dementia imaging in clinical practice: a European-wide survey of 193 centres and conclusions by the ESNR working group. Neuroradiology.

[31] Schmidt M, Schmidt SAJ, Adelborg K (2019). The Danish health care system and epidemiological research: from health care contacts to database records. Clin Epidemiol.

[32] Ellingson BM, Bendszus M, Boxerman J (2015). Consensus recommendations for a standardized Brain Tumor Imaging Protocol in clinical trials. Neuro Oncol.

[33] Schmainda KM, Prah MA, Rand SD (2018). Multisite concordance of DSC-MRI analysis for brain tumors: results of a National Cancer Institute Quantitative Imaging Network Collaborative Project. AJNR Am J Neuroradiol.

