# Supplementary material for: From research to clinical practice: a European neuroradiological survey on quantitative advanced MRI implementation
Source: Eur Radiol. 2021 Jan 22;31(8):6334–41. doi: 10.1007/s00330-020-07582-2 (PMC8270851; doi:10.1007/s00330-020-07582-2)

**Online supplement 2:** **Table 1 Demographics and structure of responding institutions**

N.B.: 27 European countries plus Russia, Turkey and Israel were successfully contacted. Greece, Poland, Slovakia and Hungary did not have any responders.

*minimum number of institutions answering, as not every professional stated his/her workplace.

n/a: return quote not available as the total number of contacted institutions and responders is not known due to contact via national neuroradiological associations only.

| Country | Radiologists contacted | Radiologists answering | Return quote | Institutions answering* | Return quote | Type of institution according to respondent working there | | | | | | Age of Respondents | | | |
| --- | --- | --- | --- | --- | --- | --- | --- | --- | --- | --- | --- | --- | --- | --- | --- |
|  |  |  |  |  |  | University Hospital | Large Hospital | Small Hospital | Practice | Tele-radiology | Research Centre | 20 - 30 | 31 - 45 | 46 - 60 | > 60 |
| Germany | 559 | 63 | 11.3% | 17 | 6.0% | 28 | 16 | 17 | 4 | 0 | 0 | 1 | 32 | 21 | 3 |
| France | 312 | 40 | 12.8% | 22 | 39.3% | 34 | 7 | 0 | 0 | 1 | 0 | 3 | 19 | 17 | 1 |
| Italy | 356 | 29 | 8.1% | 17 | 24.3% | 17 | 9 | 2 | 0 | 0 | 2 | 0 | 11 | 13 | 5 |
| The Netherlands | 256 | 27 | 10.5% | 11 | n/a | 11 | 15 | 0 | 0 | 1 | 0 | 0 | 16 | 5 | 6 |
| Russia | 1812 | 22 | 1.2% | 13 | 14.6% | 5 | 4 | 4 | 6 | 1 | 3 | 4 | 16 | 1 | 1 |
| Turkey | 528 | 14 | 2.7% | 6 | 10.2% | 14 | 0 | 0 | 0 | 1 | 0 | 0 | 8 | 6 | 0 |
| Spain | 147 | 11 | 7.5% | 6 | 15.0% | 10 | 0 | 1 | 0 | 0 | 0 | 0 | 5 | 5 | 1 |
| Switzerland | 94 | 11 | 11.7% | 5 | 12.8% | 3 | 3 | 4 | 1 | 0 | 1 | 0 | 3 | 7 | 1 |
| Denmark | n/a | 8 | n/a | 4 | n/a | 7 | 1 | 0 | 0 | 0 | 0 | 0 | 5 | 1 | 2 |
| Belgium | n/a | 8 | n/a | 4 | n/a | 2 | 4 | 2 | 0 | 0 | 0 | 0 | 3 | 3 | 1 |
| Czech Republic | 107 | 7 | 6.5% | 4 | 7.3% | 1 | 3 | 3 | 0 | 0 | 0 | 0 | 3 | 3 | 1 |
| Austria | 84 | 7 | 8.3% | 2 | 4.9% | 5 | 1 | 1 | 0 | 0 | 0 | 0 | 3 | 3 | 1 |
| Sweden | 99 | 4 | 4.0% | 3 | 25.0% | 4 | 0 | 0 | 0 | 0 | 0 | 0 | 2 | 2 | 0 |
| UK | 180 | 4 | 2.2% | 3 | n/a | 4 | 1 | 0 | 0 | 1 | 2 | 0 | 4 | 0 | 0 |
| Portugal | 17 | 4 | 23.5% | 2 | 15.4% | 3 | 2 | 0 | 0 | 0 | 0 | 0 | 2 | 1 | 1 |
| Israel | n/a | 3 | n/a | 2 | n/a | 3 | 1 | 0 | 0 | 0 | 1 | 0 | 0 | 2 | 1 |
| Ireland | 25 | 2 | 8.0% | 1 | 12.5% | 2 | 0 | 0 | 0 | 0 | 0 | 0 | 2 | 0 | 0 |
| Estonia | n/a | 2 | n/a | 2 | n/a | 0 | 1 | 1 | 0 | 0 | 0 | 0 | 0 | 2 | 0 |
| Finland/Suomi | n/a | 2 | n/a | 2 | n/a | 1 | 1 | 0 | 0 | 0 | 0 | 0 | 0 | 2 | 0 |
| Norway | 68 | 1 | 1.5% | 1 | 10.0% | 0 | 1 | 0 | 0 | 0 | 0 | 0 | 1 | 0 | 0 |
| Malta | n/a | 1 | n/a | 1 | n/a | 1 | 0 | 0 | 0 | 0 | 0 | 0 | 1 | 0 | 0 |
| Luxemburg | n/a | 1 | n/a | 1 | n/a | 0 | 0 | 1 | 0 | 0 | 0 | 0 | 0 | 0 | 0 |
| Cyprus | n/a | 1 | n/a | 1 | n/a | 1 | 0 | 0 | 0 | 1 | 0 | 0 | 1 | 0 | 0 |
| Greece | 43 | 0 | 0.0% | 0 | 0.0% | 0 | 0 | 0 | 0 | 0 | 0 | 0 | 0 | 0 | 0 |
| Poland | 54 | 0 | 0.0% | 0 | 0.0% | 0 | 0 | 0 | 0 | 0 | 0 | 0 | 0 | 0 | 0 |
| Slovakia | 12 | 0 | 0.0% | 0 | 0.0% | 0 | 0 | 0 | 0 | 0 | 0 | 0 | 0 | 0 | 0 |
| Hungary | n/a | 0 | n/a | 0 | n/a | 0 | 0 | 0 | 0 | 0 | 0 | 0 | 0 | 0 | 0 |
|  | | | | | | | | | | | | | | | |
| Total | 4753 | 272 | 6.7% | 130 | 12.3% | 156 | 70 | 36 | 11 | 6 | 9 | 8 | 137 | 94 | 25 |
|  | | | | | | 57.4% | 25.7% | 13.2% | 4.0% | 2.2% | 3.3% | 2.9% | 50.4% | 34.6% | 9.20% |

| **Sequence usage frequency (radiologists answering) by country in percent** | | | | | | | | | | | | | |
| --- | --- | --- | --- | --- | --- | --- | --- | --- | --- | --- | --- | --- | --- |
| Country  (no. of respondents) | DWI | DSC | DCE | ASL | IVIM | DKI | Fat Quant | T1 Maps | T2 Maps | MRS | PET MR | BOLD | CEST |
| Germany n=63 | 55 | 40 | 13 | 13 | 2 | 1 | 12 | 10 | 11 | 42 | 9 | 25 | 2 |
| France n=40 | 39 | 37 | 25 | 31 | 1 | 1 | 10 | 6 | 8 | 38 | 4 | 21 | 4 |
| Italy n=29 | 5 | 10 | 4 | 1 | 0 | 0 | 0 | 0 | 0 | 5 | 1 | 3 | 0 |
| Netherland n=27 | 27 | 23 | 17 | 8 | 2 | 0 | 6 | 3 | 2 | 10 | 6 | 11 | 2 |
| Russia n=22 | 16 | 7 | 12 | 5 | 2 | 1 | 5 | 4 | 4 | 5 | 0 | 5 | 1 |
| Turkey n=14 | 12 | 12 | 12 | 5 | 2 | 0 | 4 | 3 | 5 | 14 | 0 | 8 | 0 |
| Spain n=11 | 11 | 9 | 7 | 5 | 0 | 0 | 6 | 1 | 2 | 10 | 3 | 9 | 0 |
| Switzerland n=11 | 11 | 8 | 6 | 5 | 3 | 1 | 7 | 2 | 3 | 8 | 1 | 5 | 1 |
| Denmark n=8 | 7 | 4 | 3 | 2 | 0 | 2 | 1 | 0 | 0 | 5 | 1 | 1 | 0 |
| Belgium n=8 | 6 | 6 | 1 | 8 | 0 | 0 | 5 | 2 | 2 | 5 | 2 | 1 | 1 |
| Czech Republic n=7 | 7 | 3 | 5 | 3 | 0 | 1 | 3 | 4 | 4 | 5 | 1 | 4 | 0 |
| Austria n=7 | 6 | 4 | 3 | 5 | 0 | 0 | 1 | 1 | 1 | 6 | 0 | 4 | 0 |
| Sweden n=4 | 4 | 2 | 1 | 0 | 0 | 0 | 1 | 1 | 1 | 4 | 0 | 3 | 1 |
| UK n=4 | 3 | 4 | 2 | 3 | 0 | 1 | 0 | 1 | 0 | 3 | 1 | 3 | 1 |
| Portugal n=4 | 3 | 3 | 1 | 2 | 1 | 0 | 0 | 1 | 1 | 3 | 0 | 4 | 0 |
| Israel n=3 | 3 | 3 | 3 | 2 | 0 | 0 | 2 | 1 | 0 | 3 | 1 | 3 | 0 |
| Ireland n=2 | 2 | 2 | 0 | 1 | 1 | 0 | 1 | 1 | 1 | 2 | 0 | 1 | 0 |
| Estonia n=2 | 2 | 1 | 1 | 1 | 0 | 0 | 2 | 0 | 1 | 2 | 0 | 1 | 0 |
| Finland n=2 | 2 | 2 | 1 | 1 | 1 | 0 | 1 | 0 | 0 | 2 | 0 | 2 | 0 |
| Norway n=1 | 1 | 1 | 1 | 0 | 0 | 1 | 1 | 0 | 0 | 1 | 1 | 1 | 0 |
| Malta n=1 | 1 | 1 | 0 | 0 | 0 | 0 | 0 | 0 | 0 | 1 | 1 | 0 | 0 |
| Luxemburg n=1 | 0 | 0 | 0 | 0 | 0 | 0 | 0 | 0 | 0 | 0 | 0 | 0 | 0 |
| Cyprus n=1 | 0 | 1 | 0 | 1 | 0 | 0 | 0 | 0 | 0 | 1 | 0 | 1 | 0 |
| Total n=272 | 223 | 183 | 118 | 102 | 15 | 9 | 68 | 41 | 46 | 175 | 32 | 116 | 13 |

**ONLINE SUPPLEMENT 3: Q&A on questionnaire methodology and data retrieval**

This question and answer section provides several details regarding the theoretical construction of this survey.

Individual questions can be addressed to the corresponding author.

*Abbreviations: CEST=chemical exchange saturation transfer/technique, qMRI=quantitative MRI, ISMRM=International Society for MR in Medicine*

**How were the Radiologists contacted?**

The contact addresses were obtained from open national health system mailing lists or from publicly available information on department websites. This covered 4317 Radiologists in 16 countries (Russia n=1812, Germany n=559, Turkey n=528, Italy n=356, France n=312, Spain n=147, Czech Republic n=107, Sweden n=99, Switzerland n=94, Austria n=84, Norway n=68, Poland n=54, Greece n=43, Ireland n=25, Portugal n=17, and Slovakia n=12). The Radiologists received an individual invitational email sent from a dedicated university email account with a link to the questionnaire.

Radiological practices in German-speaking countries would also receive a phone call.

In the remaining countries, where direct contact details were not widely publicly available (Belgium, Cyprus, Denmark, Estonia, Finland, Israel, Luxemburg, Malta, Netherlands, and UK) national neuroradiological societies’ representatives were contacted and asked to distribute the questionnaire to their members via email including links. Through this channel, 436 Radiologists were contacted via national neuroradiological associations in the Netherlands (n=256) and the UK (n=180). Numbers of members reached in the other eight national radiological associations remain unknown.

**Why was an online survey chosen?**

This survey was designed as an online survey as this was the only way to logistically manage the sending of the data and process the incoming data. The authors are aware that a digital survey may discourage the participation of some colleagues. Additional phone calls, limited to German-speaking private outpatient institutions, were added as these institutions were usually neither contactable by personal email, nor by any professional organisation focusing on this group.

**Why did this survey avoid contacting colleagues via national or European professional organisations?**

It was assumed that doctors who are voluntary members of radiological or MRI research societies, such as the ISMRM or the European Radiological Society, ESR, have an above average interest in qMRI. This is a selection bias regarding those questions that focus on personal attitudes towards qMRI. The designers of this survey therefore chose the very intricate way to send emails to individual persons after an online search of the contact.

**Why did this survey involve Radiologists and not only Neuroradiologists?**

A recent survey identified dedicated sub-specialty training provision for Neuroradiologists in twelve European countries only [Pizzini FBT, F.; Sasiadek, M.; Ricci, P. Survey: Interventional Neuroradiology in Europe. EUMS. https://neuro.uemsradiology.eu/specialty-in-europe/#education-and-training Published 2019. Accessed 04 June 2020.]. Therefore respondents were considered as Neuroradiologists from their self-assigned or assumed daily work practice. We trusted Radiologists to correctly self-identify as Neuroradiologists based on their daily work routine and personal focus of interest.

**How were participating colleagues and centres selected?**

Between one and ten Radiologists were contacted per institution depending on availability of public addresses. During the search process, national and regional population density and a typical distribution of the medical centre types were considered to reduce selection bias. To be able to study the usage of qMRI at centres where the highest level of expertise is expected, university hospital staff were, however, deliberately overrepresented with up to 100% of university Neuroradiological units being contacted in some countries.

**Why are the numbers of colleagues contacted uneven per country?**

There are several factors contributing to this circumstance. Several factors needed consideration. Should the number of colleagues per 100,000 inhabitants be considered (“Radiologist density”), or the population of the countries, or should there be equal numbers of colleagues per country asked? Should the amount of MRI scans per year be a factor or the number of scanners? All these factors form a unique bias to a study. In the end, we chose the system presented in the question right above. There were however several limitations to our system due to restrictions in publicly available email contacts. Further, we assumed a variable probability of answering per country. In the Netherlands, e.g., there is a tightly knit network of colleagues working in academia and non-academic hospitals with experience in online surveys and interactive contributions. This is a positive predictor for high return rates. In other countries, such networking hardly exists and physicians are not used to receiving academic surveys, which implicates lower response rates. We needed to consider this by sending out a higher number of invitations where possible.

**Why were relatively many university centres contacted?**

While this survey attempted to cover the entire range of different centres per country, the authors pursued the goal to at least be able to tell with reasonable reliability how far the dissemination of qMRI has progressed in those centres that mark the spearheads of clinical innovation. These centres are frequently university hospitals. This is the reason why university centres are overrepresented in the survey. This technique of examining subgroups with different intensity is known as “stratified sampling”.

**Why were some European countries not part of this survey?**

Several countries (Croatia, Bosnia-Hercegovina, Serbia with Kosovo, Montenegro, Ukraine, Moldova, Bulgaria, Northern Macedonia, Albania, Latvia, Lithuania and Romania) could not be included due to language barriers on our side affecting the search method for contacts and/or lack of contact to the respective National organisations. For several countries neither a direct email contact, nor a contact via a society could be established.

**How come some countries have very low return rates?**

Several factors may contribute. First, it might be unusual to participate in surveys, especially foreign surveys, in some countries. Second, some recipients received this survey in English despite this being not their native language. This can be a discouraging factor. Third, the workload may vary between countries. We know e.g. from the UK that their number of specialists is extremely low compared to other European countries (4/100,000 inhabitants, which is one third of the EU average). A high workload may hinder potential participants from answering.

**Why are some quantitative techniques not explicitly asked for?**

This survey covered a great many techniques and included a possibility to state additional techniques in open answer spaces (compare supplement 1). However, the authors decided to limit the number of explicitly explored techniques as too many answer options can deter the participant from continuing the questionnaire or implicitly encourage the participant to give false/arbitrary answers out of distraction. The techniques included were determined by vote and after discussion of four co-authors.

**Why were small centres added in the first place? Does their duty not lie elsewhere?**

It depends on the respective country in how far smaller practices need to cover advanced medical care, too. There is not everywhere a hierarchy between centres. Also, this survey explicitly wanted to find out how advanced trickle-down effects worked for ach technique. Therefore also small centres needed to be asked whether they used very experimental techniques such as CEST despite a likeliness to receive a 0% positive reply rate.

**Is this survey representative?**

Assuming an average of 12 Radiologists per 100,000 inhabitants in the EU and around 800 million people living in Europe including Turkey and Israel, on can expect about 96,000 Radiologists, of whom a fraction is working in Neuroradiology. We contacted 4753 of these of whom a bit more than 5% answered. This is not a low return quote. Our survey contains answers of 272/96,000=0.28% of all Radiologists in extended Europe. This may conservatively be extended to 1%, assuming that one in four of all Radiologists is involved in some kind of neuroradiological duties, as neuroradiology is a large part of the field. We may further consider that one respondent per institution covers representatively the entire institution concerning all questions dealing with usage dissemination. Therefore the number of colleagues reached is indirectly higher. In comparison, a representative National poll asking about the election-winning party of a country like Germany would comprise 2,000 respondents in a country of 61,6 million potential voters covering thus 0.003%.

This non-representative aspect is founded in the unequal response rates per country, which is partially based on unequally distributed questionnaires for the abovementioned reasons. This is a clear limitation to this, albeit unique survey, on a local subgroup level.


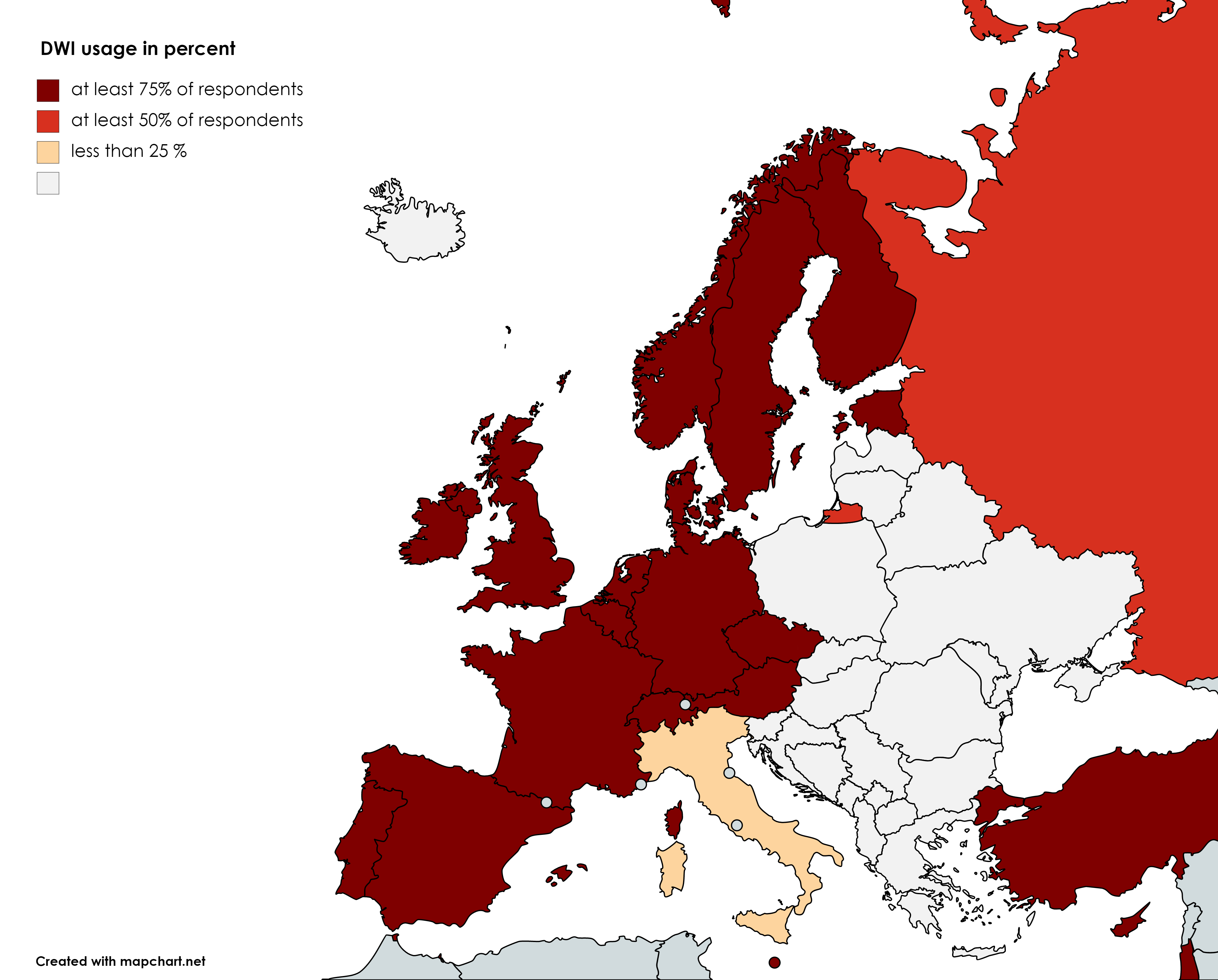


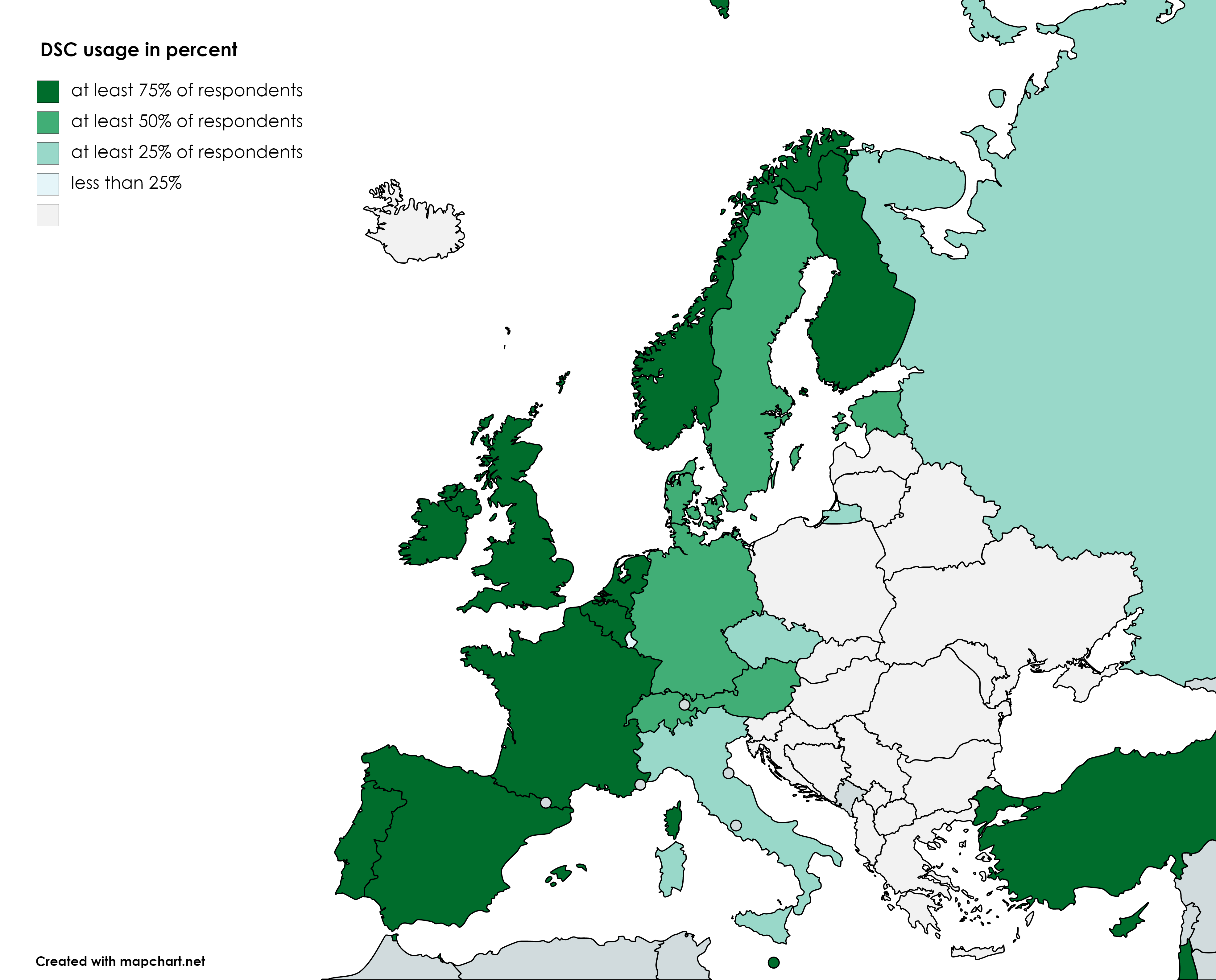


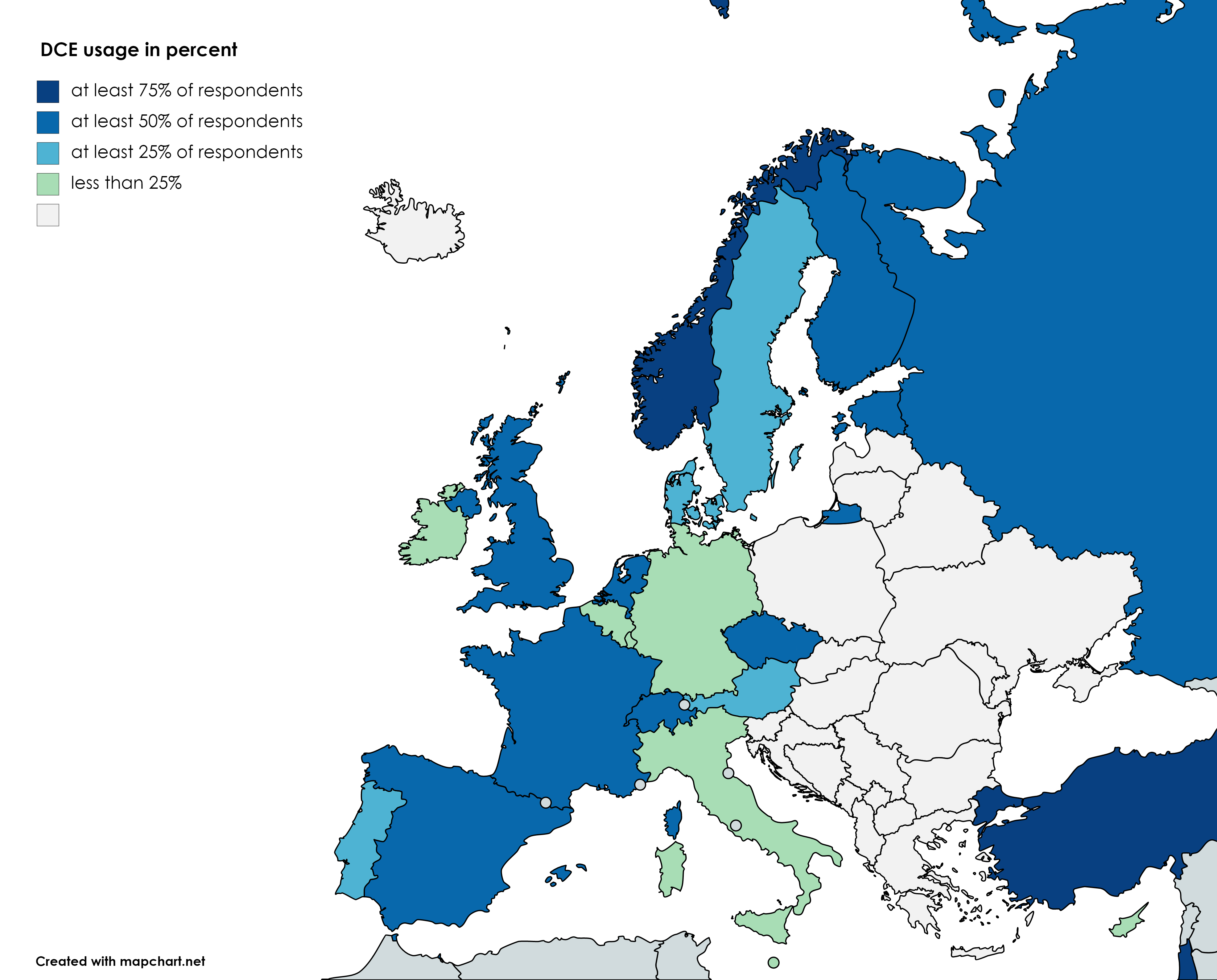


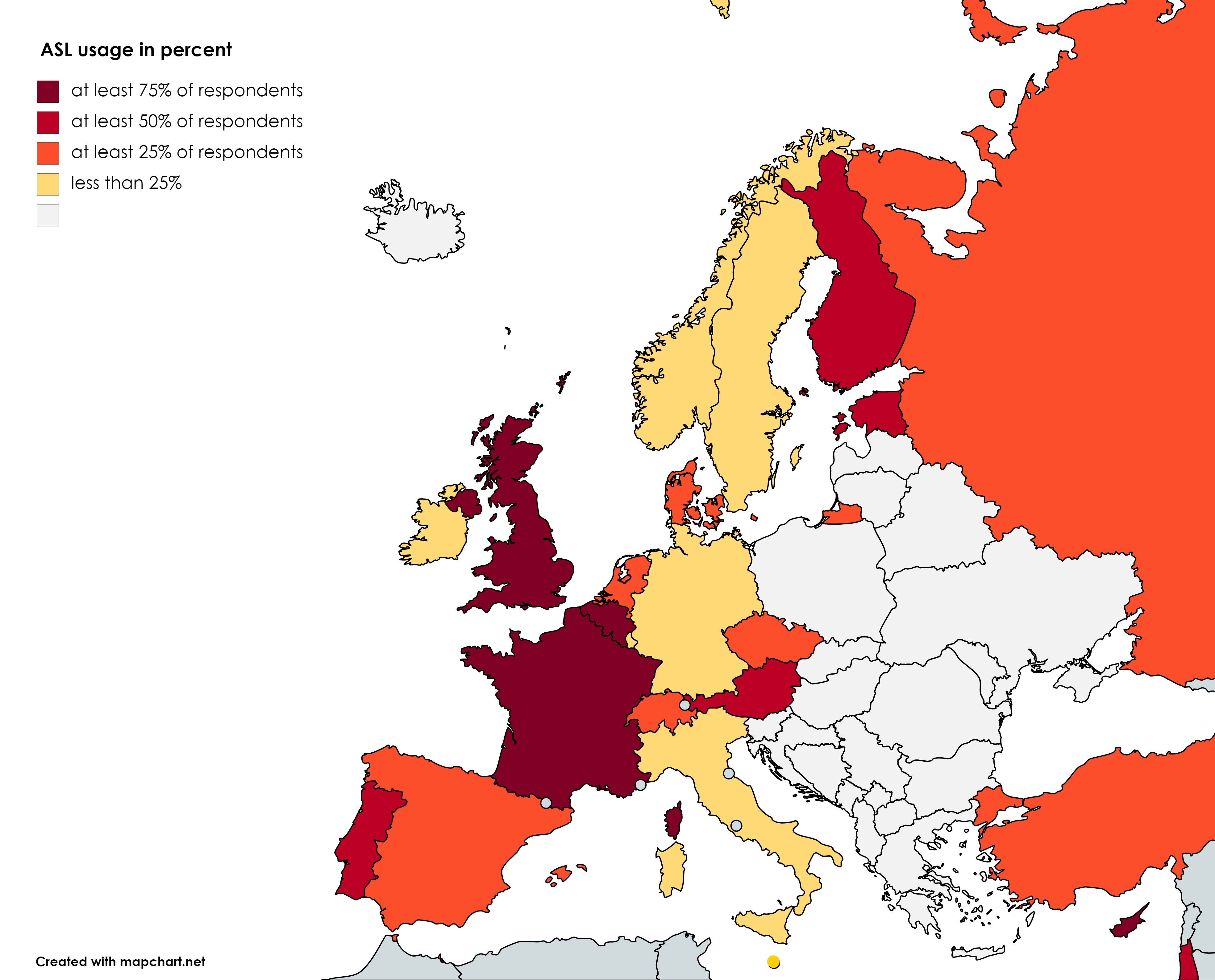


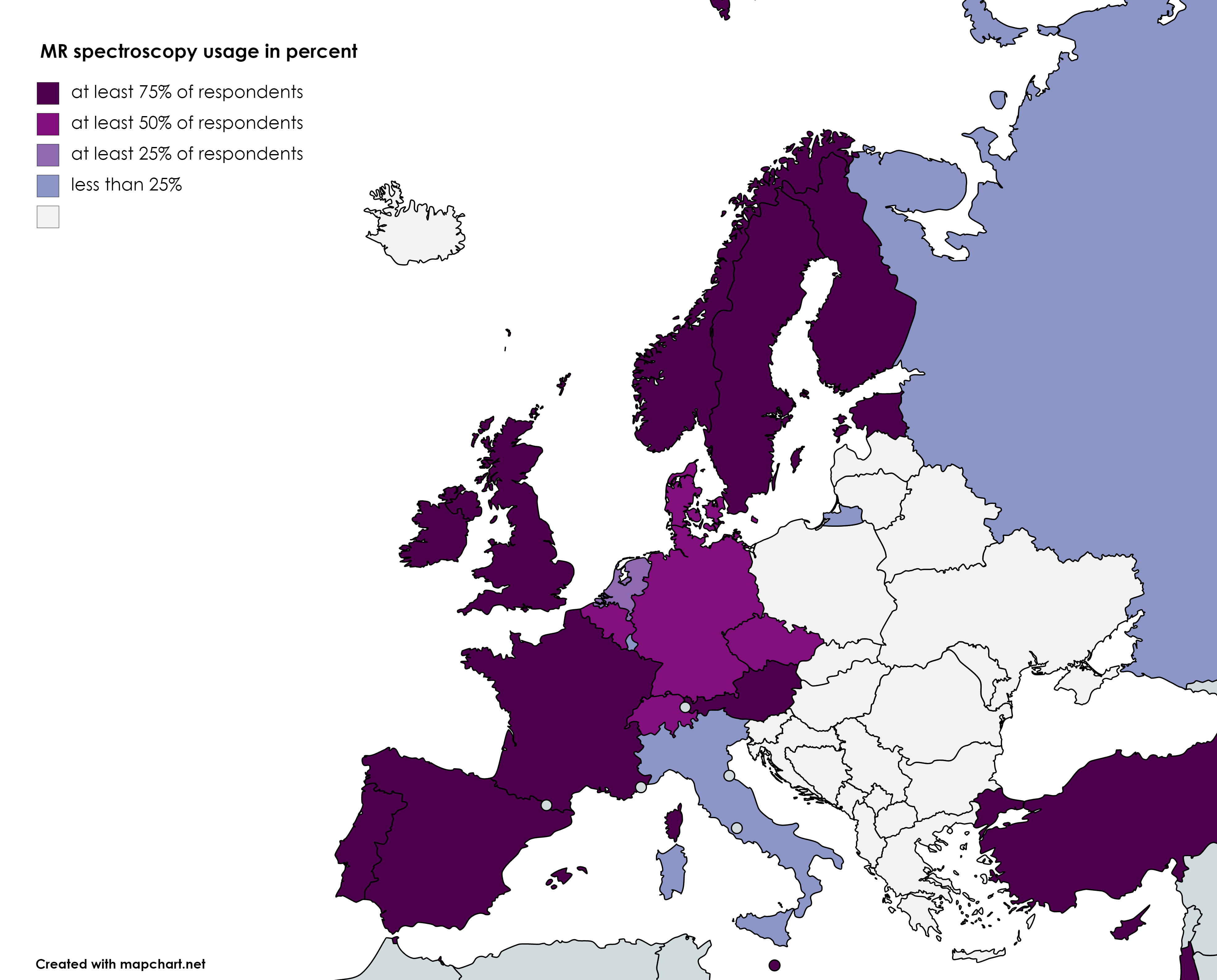

Supplement: Supplementary file 1 — (DOCX 8445 kb) [file 330_2020_7582_MOESM1_ESM.docx]
